# Non-Robotic Endoscopic-Assisted Internal Mammary Artery Harvest—A Historical Review and Recent Advancements

**DOI:** 10.3390/jcdd12020068

**Published:** 2025-02-13

**Authors:** De Qing Görtzen, Fleur Sampon, Joost Ter Woorst, Ferdi Akca

**Affiliations:** Department of Cardiothoracic Surgery, Catharina Hospital, 5623 EJ Eindhoven, The Netherlands; deqing.gortzen@catharinazieken.nl (D.Q.G.); fleur.sampon@catharinaziekenhuis.nl (F.S.); joost.t.woorst@catharinaziekenhuis.nl (J.T.W.)

**Keywords:** endoscopic harvest, thoracoscopic, video-assisted, VATS, internal mammary artery

## Abstract

*Background*: The non-robotic endoscopic harvest of the internal mammary artery (IMA) facilitates minimally invasive bypass grafting while minimizing chest wall trauma. The technique was pioneered in the early 1990s and has recently regained popularity due to its accessibility and reproducibility. This review aims to provide an overview of endoscopic IMA harvest from its inception to the present. *Methods*: In August 2024, a literature search was performed using the electronic databases of the Cochrane Controlled Trials Register (CCTR) and PubMed. To obtain optimal search results, the keywords “*thoracoscopic*”, “*endoscopic*”, “*minimally invasive*”, “*video-assisted*”, “*video-assisted thoracoscopic surgery VATS*”, and “*internal mammary artery*” or “*internal thoracic artery*” were used, excluding the term “*robotic*”. References from the extracted articles were also reviewed to identify additional studies on endoscopic IMA harvest. *Results*: A total of 17 articles were included in the final analysis. Left internal mammary artery (LIMA) harvest times of between 17 and 164 min were reported, with an injury to LIMA rates between 0.7 and 2.2%. *Conclusions*: After a 15-year period without scientific publications, interest in the endoscopic-assisted approach has rekindled in recent years due to the reduction in chest trauma compared to direct vision harvest and the widespread availability of conventional endoscopic tools. This renewed focus underscores the potential to make minimally invasive coronary surgery available in all centers.

## 1. Introduction

In coronary bypass surgery, the left internal mammary artery (LIMA) is widely endorsed as an ideal graft conduit due to the higher long-term patency rates. Studies have demonstrated the LIMA’s superior durability rates compared to venous grafts, making them the preferred option for coronary revascularization [1]. Subsequently, the LIMA to left anterior descending (LAD) artery anastomosis is considered the optimal revascularization configuration for the anterior wall [2].

Traditionally, the LIMA can be harvested through median sternotomy using an open approach. The open approach provides direct access, but surgical trauma caused by the required median sternotomy poses a risk of deep sternal wound infections and longer post-operative recovery [3]. In the mid- to late-1990s, researchers in the pursuit of less invasive techniques conducted feasibility studies in canines to determine whether a video-assisted endoscopic harvest of the IMA was possible [4]. Soon after, the first endoscopic harvests of IMAs were successfully harvested in humans. However, despite these promising early results, safety concerns regarding the steep learning curve compared to a sternotomy approach limited the technique’s widespread adoption [5].

Initially, the focus was placed on non-robotic endoscopic LIMA harvesting, but over time, robotic-assisted endoscopic techniques became standard practice. Robotic endoscopic surgery has the advantage of three-dimensional visualization and tool-manipulated stability. However, the acquisition of a robotic surgery unit is expensive, and the operation time greatly increases [6]. The endoscopic LIMA harvest technique using conventional endoscopic surgery tools became a niche, with initially only a few surgeons performing this technique. Recently, the potential for a more accessible and cost-effective option in minimally invasive coronary surgery has rekindled interest. This review aims to provide a comprehensive overview of the endoscopic LIMA harvest techniques and outcomes.

## 2. Materials and Methods

### 2.1. Literature Search

**Figure 1:**
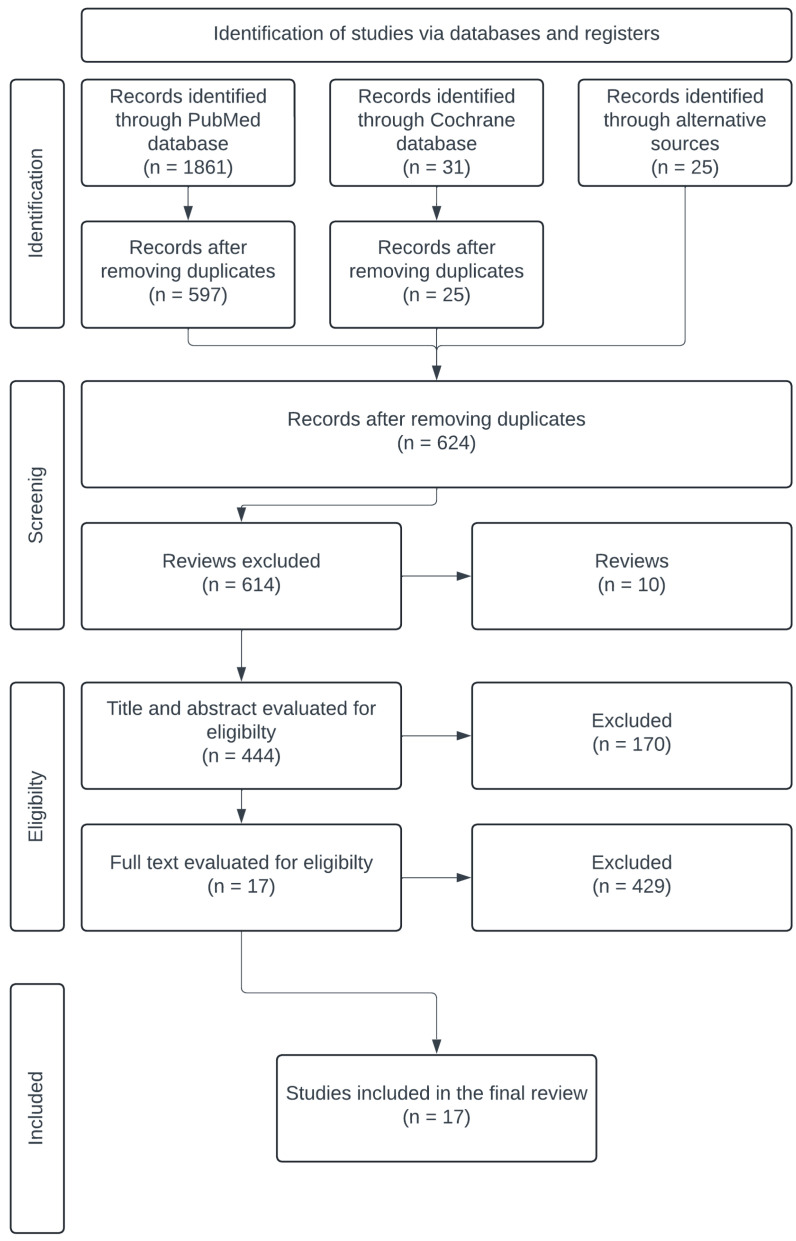
The search strategy, illustrated using the PRISMA diagram. A total of 17 articles were selected for final analysis.

In August 2024, a literature search was carried out using the electronic databases of Cochrane Controlled Trials Register (CCTR) and PubMed. To extract the optimal search results: “*thoracoscopic*”, “*endoscopic*”, “*minimally invasive*”, “*video-assisted*”, “*VATS*”, and “*internal mammary artery*” or “*internal thoracic artery*” as either keywords or Medical Subject Headings (MeSH) terms. The term “*robotic*” was specifically excluded. Furthermore, references to these articles were searched for additional results on endoscopic IMA harvesting. A manual review of these results was performed to ensure that all eligible articles were included. The search strategy is shown in the PRISMA diagram in Figure 1.

### 2.2. Study Selection

Eligible articles for this literature review were full-length cohort studies describing nonrobotic endoscopic/thoracoscopic/video-assisted IMA harvest. Studies that did not provide perioperative data on IMA harvest were excluded. Articles describing IMA harvest under direct vision through a mini-thoracotomy were also excluded. Conference presentations, how-to articles, and reviews were also excluded. The full inclusion and exclusion criteria are shown in Table 1.

## 3. Results

**Figure 2:**
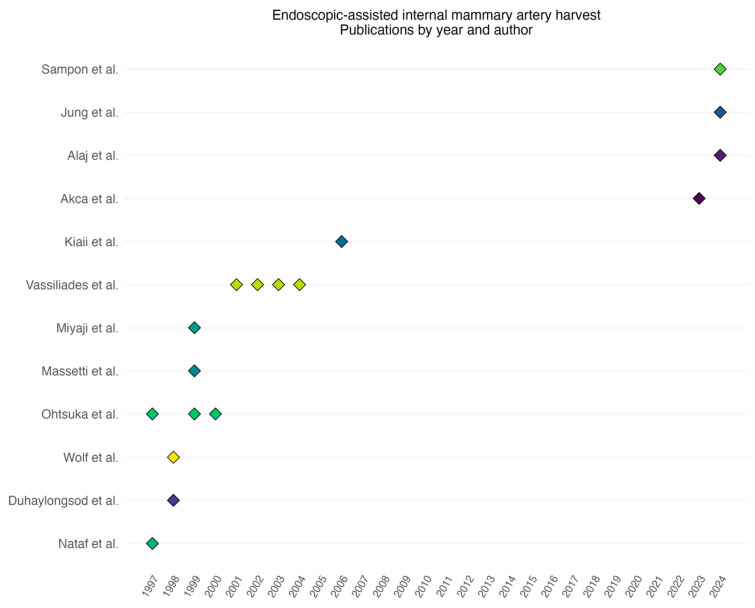
Timeline of annual publications per author, highlighting a 15-year gap with no scientific publications related to endoscopic internal mammary artery (IMA) harvest.

A total of 17 articles were included in the final analysis, which were published between 1997 and 2023. Nataf et al. published the first article concerning perioperative LIMA harvest outcomes in 1997 [7]. After this first publication, there was a surge of interest in endoscopic LIMA harvesting over a period of 9 years, with a total of 12 publications concerning the endoscopic LIMA harvest. However, in the years between 2007 and 2022, no scientific data were published on the topic of endoscopic LIMA harvest, until a study by Akca et al. [8] was published in 2023 (Figure 2 and Table 2).

As shown in Table 3, the demographics of the patients demonstrate a predominance of men and a mean age of 56.0 to 71.5 years. LIMA harvest times between 17 and 164 min were reported, with an injury to LIMA rates between 0.7 and 2.2%, as shown in Table 4. In an early publication by Duhaylongsod et al., there was a reported conversion rate to open median sternotomy of 8.3%, with subsequent publications reporting declining conversion rates of between 0.7 and 3.8% [9,10,11,12]. Postoperative results are shown in Table 5. A total hospital stay between 2.3 and 6 days was reported after minimally invasive coronary artery bypass surgery using endoscopic-assisted IMA harvest. Transfusion rates between 2.2% and 15.0% and new-onset postoperative fibrillation rates between 2.9% and 21.6% were reported. Postoperative myocardial infarction rates ranged between 0.7% and 2.3%.

**Table 2:** List of studies of non-robotic endoscopic LIMA harvest included in the literature review.

Study	Year of Publication	Study Period	Type of Study	Number of Patients	Single or Multivessel
Nataf [7]	1997	1995–1996	Retrospective	32	Single
Ohtsuka [13]	1997	1995–1996	Retrospective	37	Both
Wolf [14]	1998	1995–1997	Retrospective	48	Both
Duhaylongsod [9]	1998	1995–1997	Retrospective	218	Both
Ohtsuka [15]	1999	1997–1998	Retrospective	22	Single
Miyaji [16]	1999	1993–1998	Retrospective	73	Both
Massetti [17]	1999	1996–1997	Retrospective	30	Single
Ohtsuka [18]	2000	1997–1999	Retrospective	38	Both
Vassiliades [19]	2001	1996–2001	Retrospective	300	Both
Vassiliades [20]	2002	1996–2001	Retrospective	350	Both
Vassiliades [21]	2003	2002–2002	Retrospective	18	Multivessel
Vassiliades [11]	2004	1996–2003	Retrospective	509	Both
Kiaii [22]	2006	NR	Prospective	50	Single
Akca [8]	2023	2021–2022	Retrospective	80	Single
Jung [23]	2024	2019–2023	Retrospective	40	Both
Alaj [24]	2024	2021–2022	Retrospective	91	Both
Sampon [10]	2024	2018–2023	Retrospective matched cohort	266	Single

## 4. Discussion

### 4.1. Endoscopes Used During LIMA Harvest

Depending on the surgeon’s preference, different endoscopic tools can be used during the LIMA harvesting process. In the years after the technique was initially described, different rigid video endoscopes (0 degrees and 30 degrees) were explored to investigate which provided the best vision during the harvesting process. In the first publications of the surgical technique in humans, both 0-degree and 30-degree endoscopes were used. Some articles reported that the LIMA may be difficult to visualize due to obstruction by the cardiac mass at the fourth and fifth intercostal spaces. A 30-degree endoscope was introduced, in order to improve visualization [13,25]. Tevaearai et al. suggested a modified flexible gastroscope to improve visualization, since the movement axis of a rigid video endoscope is the same as the dissecting scalpel. Furthermore, the visualization of the LIMA becomes more difficult during harvesting of the distal parts, and a flexible gastroscope could mitigate this and minimize unnecessary surgical instruments in the surgical field [26]. In more recently published studies, a 0-degree (3D) endoscope has been used during the harvesting process, in order to provide a full visualization of the LIMA during harvest [8,23,24]. Another factor is the assistant, who is needed to adjust the video endoscope during the LIMA harvest. Since it is essential that the surgeon always has a good visualization of the LIMA during the harvesting process, precise instruction concerning the placement of the camera needs to be given to the assistant. Vassiliades et al. described the technique of using a voice-activated endoscope to perform the harvest without a surgical assistant [19].

### 4.2. Energy Source During LIMA Harvest

During endoscopic IMA harvest, a conventional electrocautery scalpel (EC), Ligasure Maryland device (Medtronic, Dublin, Ireland), or a harmonic scalpel (HS) can be used. The first endoscopic IMA harvest in a human was accomplished in 1994, using a prototype of the harmonic scalpel with a hook blade, by Randall K. Wolf [13]. For endoscopic IMA harvesting, all types of scalpel are used [8,23,24]. A large meta-analysis by Kaneyuki et al., comparing HS to EC, reported the slowest recorded LIMA harvesting times with HS. They theorized that increased LIMA harvest times were caused by a higher rate of skeletonized harvesting in the HS group. There were no significant differences in postoperative outcomes between the two techniques [27]. Jung and colleagues studied a shear-tip harmonic scalpel for endoscopic, clipless IMA harvest [23]. A difficulty in managing bleeding or damage during endoscopic IMA harvest using EC can lead to higher conversion rates to sternotomy [23]. The Ligasure vessel sealing device has been described for both pedicled and skeletonized IMA harvest previously, with adequate ligation of side branches demonstrated [8,10,23].

### 4.3. Pedicled or Skeletonized Harvest

IMA can be harvested as either a skeletonized or pedicled graft. Traditionally, the IMA is harvested as a pedicled graft containing the IMA, veins, and fascia. The technique to harvest the IMA in a skeletonized manner is technically more challenging and has a higher chance of IMA damage [28]. A review of pedicled versus skeletonized techniques demonstrated that the advantages of skeletonized harvesting include a longer graft conduit length and improved IMA flow [29]. In the studies included in this review, it was observed that, in the early days of endoscopic IMA harvest, the IMA was harvested as a pedicled graft [7,13,25]. Considering the steep learning curve associated with endoscopic IMA harvest and the available endoscopic technology at the time, a pedicled harvest is understandable. In recent years, however, after having overcome most technical obstacles, surgeons are electing to harvest the skeletonized IMA, a procedure with associated benefits, as described above [8,10,23].

### 4.4. Overview of the General Technique of Endoscopic LIMA Harvesting

**Figure 3:**
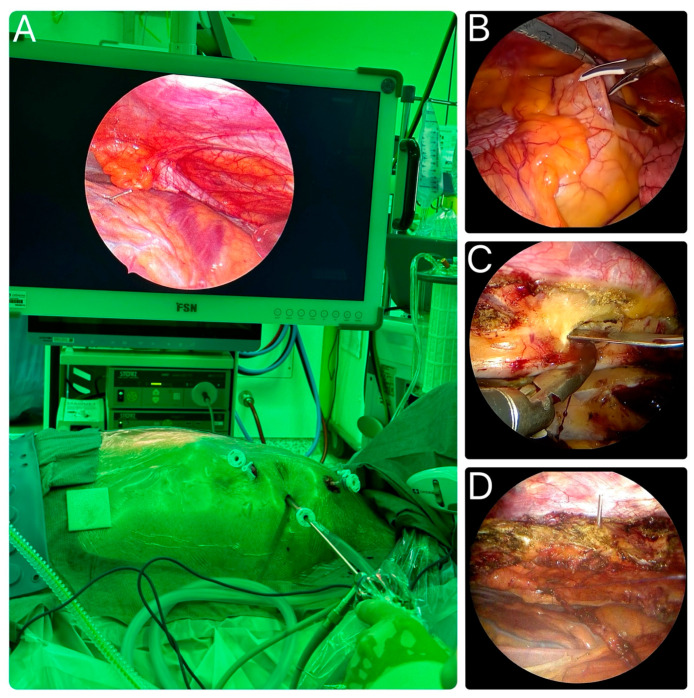
(**A**) Endoscopic left internal mammary artery (LIMA) harvest setup, as demonstrated by Akca [8]. (**B**) Visualization of coronary targets on the video monitor following the opening of the pericardium. (**C**) Endoscopic LIMA harvest using the Ligasure scalpel. (**D**) Needle-guided marking of the mini-thoracotomy technique, as described by Ohtsuka [31].

After discussing the various approaches that can be used for endoscopic IMA harvest, a summary of the procedure is provided here. Once general anesthesia is administered, the patient is placed in a supine position. Often, an object such as a pillow is placed under the left scapula to elevate the left hemithorax. Depending on the surgeon’s preference, the arm on the side of the harvest is either kept to the side of the thorax or is positioned above the head of the patient to provide better access to the lateral chest wall. The ports are placed around the third, fifth, and seventh intercostal spaces between the mid and anterior axillary lines. The exact location of the mini-thoracotomy can differ per patient due to anatomical differences and the location of the anticipated left anterior small thoracotomy (LAST) for the anastomoses. Carbon dioxide insufflation, at levels of 8 to 10 mm, facilitates the LIMA harvest, with ventilated lungs or single lung ventilation. A 0- or 30-degree video endoscope and standard endoscopic tools are introduced. The LIMA can be readily visualized adjacent to the internal thoracic vein on the video monitor. The LIMA can be harvested in a pedicled or (semi)skeletonized fashion. The phrenic nerve can be easily identified proximally. The side branches are clipped or ligated, depending on the tools used during the LIMA harvest. Both the left and right IMA can be harvested using this technique for multivessel total arterial revascularization. The radial artery can also be harvested simultaneously as a second graft conduit, depending on the surgeon’s preference. Once the LIMA has been harvested entirely, heparin is administered and the LIMA is divided for the coronary anastomosis [9,21,30]. Figure 3 demonstrates the setup of the endoscopic LIMA harvest by Akca et al., as well as different stages of the harvesting process, which are highlighted [8].

### 4.5. Patient Selection and Post-Operative Results

The commonly reported patient exclusion criteria for endoscopic IMA are chest radiation or trauma, emergency operations, and hemodynamically unstable patients. Certain anatomical conditions increase the technical difficulty of the endoscopic IMA harvest, such as morbid obesity, pectus excavatum, or severe scoliosis. Vassiliades et al. describe difficulties in positioning patients with morbid obesity and challenges in endoscopic IMA harvest. However, no adverse post-surgical outcomes have been reported compared to patients with normal BMI [20]. Redo procedures or left subclavian artery stenosis are generally also (relative) contraindications. Differences between male and female anatomy should also be taken into consideration regarding avoiding the breast tissue for a more aesthetically pleasing result. The incidence of post-operative infections seen with sternal incisions is also avoided by using a thoracoscopic approach. Certain patient populations benefit especially from the endoscopic approach. Patients with high-risk baseline characteristics, such as diabetic patients, those with obstructive pulmonary disease, and elderly patients, benefit from a minimally invasive approach due to a shorter hospital stay and reduced rates of post-operative infection [32,33]. Excellent mid-term patency rates are reported in two studies; Miyaji et al. and Kiaii et al. report 6-month graft patency rates of 97.2% and 98.0% [16,22].

### 4.6. Cost and Availability of Materials

In the years after the first endoscopic IMA harvest, robotic IMA harvest quickly increased in popularity and there is constant innovation in robotic techniques. However, a cost-effective, accessible, and reproducible technique has benefits for the popularization of minimally invasive coronary surgery. Vassiliades et al. reported in 2001 that the procedural costs for endoscopic IMA harvest were lower than those for robotically assisted total endoscopic coronary artery bypass (TECAB). Direct-vision IMA harvest was reported as having the lowest cost [19]. Similarly, Akca et al. reported the added benefits of using conventional endoscopic tools in minimally invasive coronary surgery, making it more available for centers that do not have access to a robot [8].

## 5. Conclusions

Endoscopic IMA harvest was first described in the late 1990s and continues to hold a strong position in minimally invasive coronary surgery. After a period between 2007 and 2022 without scientific publications, interest in this approach has rekindled in recent years due to the reduction in chest trauma compared to direct vision harvest and the widespread availability of conventional endoscopic tools. This renewed focus underscores the potential to make minimally invasive coronary surgery available in all cardiac surgery centers.

## Figures and Tables

**Table 1:** Inclusion and exclusion criteria.

Inclusion Criteria	Exclusion Criteria
Internal mammary harvest (IMA)	Reviews
Thoracoscopic-assisted	Direct-vision harvest
Video-assisted	Robotic harvest
Endoscopic	Animal training models
Perioperative data about IMA harvest	Other thoracic surgery

**Table 3:** Study demographics.

Study	Age (Years)	Sex (Men)	Chronic Obstructive Pulmonary Disease (COPD)	Previous Cardiac Surgery
Nataf [7]				
Ohtsuka [13]	70.2 [47–89]	13 (56.5)		
Wolf [14]				4 (8.7)
Duhaylongsod [9]	61.7 [38–89]	170 (78.0)		4 (1.8)
Ohtsuka [15]	71.5 ± 6.5	18 (81.8)		
Miyaji [16]	64.0 ± 12.1	43 (58.9)	11 (10.0)	7 (7.7)
Massetti [17]	67.0 ± 10.0	18 (60.0)		
Ohtsuka [18]	69.5 ± 11.5	32 (84.2)		
Vassiliades [19]	69.8 [28–85]	191 (63.7)		11 (3.7)
Vassiliades [20]	56.0 ± 11.0	219 (62.6)		
Vassiliades [21]	58.9 ± 13.1	12 (66.7)		
Vassiliades [11]	64.5	345 (67.7)	136 (26.8)	
Kiaii [22]	56.9 ± 11.2	44 (88.0)		
Akca [8]	66.0 ± 9	64 (79.0)		
Jung [23]	70.0 [30–86]	27 (67.5)	3 (7.7)	
Alaj [24]	65.1 ± 10.1	79 (86.8)	9 (9.9)	0
Sampon [10]	64.0 [58–70]	111 (81.6)	3 (2.2)	

**Table 4:** Surgical parameters.

Study	Tool	Skeletonized/Pedicled	LIMA Time (min)	LIMA Flow (mL/min)	Right Internal Mammary Artery (RIMA) Time (min)	RIMA Flow	Total Operation Time	Conversion Rates	Injury to LIMA
Nataf [7]	EC	Pedicled	58.7 [20–130]					0	0
Ohtsuka [13]	HS	Pedicled	42 [28–48]		28			0	0
Wolf [14]	HS	Pedicled	65 [35–95]	20–110	37 [25–45]			0	1 (2.2)
Duhaylongsod [9]	EC	Pedicled	48 [29–95]		29 [25–45]			18 (8.3)	4 (1.8)
Ohtsuka [15]	HS	Pedicled	44 ± 12					0	
Miyaji [16]	HS	Pedicled		31.2 ± 12.4					
Massetti [17]	EC	Pedicled	90						
Ohtsuka [18]	HS	Pedicled	40.8 ± 12.2		33.5 ± 8.5			0	
Vassiliades [19]	EC	Pedicled	24.4 [17–61]				96.4 [54–154]	0	2 (0.7)
Vassiliades [20]	EC	Pedicled	37.6 ± 12		37.6 ± 12		126 ± 36	9 (2.6)	
Vassiliades [21]	EC	Pedicled	52.3 ± 17.5	61.1 ± 13.0	35.6 ± 6.7	56.4 ± 14.1	211 ± 14	0	0
Vassiliades [11]	EC	Pedicled						20 (3.8)	0
Kiaii [22]	HS	Pedicled	63.3 ± 20.3	33.7 (19.3)		[35–95]		0	0
Akca [8]	Ligasure	Skeletonized	58 ± 19	41 ± 25			150 ± 39	0	0
Jung [23]	HS	Skeletonized	87 [25–164]	22 [5–73]	24 [19–50]	22.3 [17–30]		0	
Alaj [24]	EC	Pedicled					156 ± 48	0	
Sampon [10]	Ligasure	Skeletonized	48 [37–61]				125 [104–150]	1 (0.7)	

**Table 5:** Post-operative results.

Study	Intensive Care Unit (ICU) Stay (Hours)	Total Hospital Stay (Days)	Transfusion	Atrial Fibrillation (AF) de Novo	30-Day Mortality	Myocardial Infarction (MI)	Cerebrovascular Accident (CVA)	Wound Reintervention	Pneumonia	Phrenic Nerve Injury
Nataf [7]										
Ohtsuka [13]										0
Wolf [14]					1 (2.2)					1 (2.2)
Duhaylongsod [9]					5 (2.3)		1 (0.5)	6 (2.8)		1 (0.5)
Ohtsuka [15]										
Miyaji [16]	29.0 ± 20.5	4.2 ± 2.1		2 (2.9)	2 (2.9)			2 (2.9)		
Massetti [17]										
Ohtsuka [18]										
Vassiliades [19]	11.9	2.4	32 (10.5)	65 (21.6)	1 (0.3)	3 (0.7)	3 (0.7)	4 (1.3)	43 (14.3)	
Vassiliades [20]	5.23 ± 4.33	2.3 ± 1.2	30 (11.7)		4 (1.4)	8 (2.3)		6 (1.7)	4 (1.3)	
Vassiliades [21]	6.9 ± 4.5	2.3 ± 0.3			0					
Vassiliades [11]	40.8	5.25	77 (15.0)	102 (20.0)	0	0	0	0		
Kiaii [22]	17.8 ± 8.5			2 (4.0)	0	0	0			
Akca [8]										
Jung [23]	23.0	6 [3–22]	1 (2.5)		0			2 (5.0)	1 (2.5)	
Alaj [24]	36.0 ± 38.4				0	1 (1.0)	0	2 (2.2)		
Sampon [10]	12 [12–24]	3.0 [3.0–4.0]	3 (2.2)	10 (7.4)	1 (0.7)	1 (0.7)		1 (0.7)	0	1 (0.7)

## Data Availability

Data will be made available upon reasonable request.

## Abbreviations

CABG = coronary artery bypass grafting; LIMA = left internal mammary artery harvesting; LAD = left anterior descending; IMA = internal mammary artery; LAST = left anterior small thoracotomy; MeSH = Medical Subject Headings; COPD = Chronic obstructive pulmonary disease; RIMA = Right internal mammary artery; ICU = Intensive care unit; AF = Atrial fibrillation; MI = Myocardial infarction; CVA = Cerebrovascular accident; HS = harmonic scalpel; EC = conventional electrocautery; TECAB = totally endoscopic coronary artery bypass.
